# Genetic Variants Associated With Subjective Cognitive Decline in Patients With Migraine

**DOI:** 10.3389/fnagi.2022.860604

**Published:** 2022-06-15

**Authors:** Po-Kuan Yeh, Chih-Sung Liang, Chia-Lin Tsai, Yu-Kai Lin, Guan-Yu Lin, Chia-Kuang Tsai, Ming-Chen Tsai, Yi Liu, Yueh-Ming Tai, Kuo-Sheng Hung, Fu-Chi Yang

**Affiliations:** ^1^Department of Neurology, Tri-Service General Hospital, National Defense Medical Center, Taipei, Taiwan; ^2^Department of Psychiatry, Beitou Branch, Tri-Service General Hospital, National Defense Medical Center, Taipei, Taiwan; ^3^Department of Neurology, Songshan Branch, Tri-Service General Hospital, National Defense Medical Center, Taipei, Taiwan; ^4^Center for Precision Medicine and Genomics, Tri-Service General Hospital, National Defense Medical Center, Taipei, Taiwan

**Keywords:** migraine, subjective cognitive decline, comorbidity, single nucleotide polymorphisms, genetic variants

## Abstract

The genetic association between subjective cognitive decline (SCD) and migraine comorbidity remains unclear. Furthermore, single nucleotide polymorphisms (SNP) associated with SCD have not been identified previously. Migraineurs were genotyped using an Affymetrix array. The correlation between different SNP variants in migraineurs with or without SCD and non-migraine controls was investigated. Migraineurs with or without SCD were further divided for the analysis of relevant SNP variants linked to migraine with aura (MA), migraine without aura (MoA), episodic migraine (EM), and chronic migraine (CM). Significant connectivity between SNPs and clinical indices in migraineurs and non-migraine controls with SCD were assessed using multivariate regression analysis. The rs144191744 SNP was found in migraineurs (*p* = 3.19E-08), EM (*p* = 1.34E-07), and MoA(*p* = 7.69E-07) with and without SCD. The T allele frequency for rs144191744 in TGFBR3 was 0.0054 and 0.0445 in migraineurs with and without SCD (odds ratio, 0.12), respectively. rs2352564, rs6089473 in CDH4, rs112400385 in ST18, rs4488224 and rs17111203 in ARHGAP29 SNPs were found, respectively, in non-migraineurs (*p* = 4.85E-06, *p* = 8.28E-06), MoA (*p* = 3.13E-07), and CM subgroups (*p* = 1.05E-07, 6.24E-07) with and without SCD. Rs144191744 closely relates to SCD with the all-migraine group and the EM and MoA subgroups. In conclusion, rs144191744 in TGFBR3 was significantly associated with SCD in migraineurs, especially in the EM, MoA, and female patient subgroups. Furthermore, three SNPs (rs112400385, rs4488224, and rs17111203) were associated with SCD in migraineurs but not in non-migraine controls.

## Introduction

Migraine, a common and incapacitating neurovascular disorder among patients visiting neurology clinics, has an estimated worldwide prevalence of 10%–20% and a 2–3:1 female-to-male predominance (Lipton et al., 2007). Migraine incurs wide-ranging effects, negatively impacting work productivity and career progression, social and family interactions, and health-related quality of life (Stewart et al., 2001). Some individuals will experience an aura prior to migraine onset in the form of flashes, abnormal smells, unusual body feelings, or gastrointestinal discomfort (Goadsby et al., 2017). Some patients report that their primary cause of disability is not their pain but their symptoms, such as cognitive impairment.

Subjective cognitive decline (SCD), also known as subjective memory complaints, like difficulty naming things and recalling the position of something, despite normal performance on objective neuropsychological tests, are frequent among the general adult population (10.4%–18.8%; Taylor et al., 2018), and the prevalence increases with age. SCD may be associated with normal aging, personality characteristics, and neuropsychiatric disorders (Mendonca et al., 2016). These older individuals with SCD who may or may not have deficits on objective testing (Mitchell et al., 2014) and individuals with mental illnesses, such as depression and anxiety, are usually excluded from clinical studies. However, additional cognitive impairment and progression to dementia are still frequent in older individuals with SCD. In community research, memory impairment was an important predictor of impending dementia. Young people with subjective cognitive problems have a higher risk of dementia than older individuals (Jonker et al., 2000). The National Institute on Aging and Alzheimer’s Association revised their research guidelines for Alzheimer’s disease (AD). They defined SCD as a probable clinical stage 2 in the Alzheimer’s continuum, which exhibits normal performance within expected ranges on objective cognitive evaluations, a distinctive transitional stage between asymptomatic (Stage 1) and symptomatic mild clinical impairment (MCI; Stage 3; Jack et al., 2018). However, the pathophysiology remains largely unknown.

The underlying pathogenesis of SCD remains largely unknown. Previous research has built an association between late-onset AD and the Apolipoprotein E (*APOE*) gene, especially rs429358 in APOE4 (Liu et al., 2015) and rs7412 in APOE2 (Chen, 2016). Currently, there is no clear, established relationship between the *APOE* gene and SCD. Early-onset AD is related to mutations in the three familial AD genes, namely amyloid precursor protein (APP, multiple mutations such as 670/671 Swedish mutation for favoring β-secretase cleavage, 717 transmembrane domain mutation for overproduction of Aβ42, A692G Flemish/E693Q Dutch, and p. E693G Arctic mutations for facilitating Aβ protofibril formation) on chromosome 21q21.3, presenilin 1 (PSEN1, S182) on chromosome 14q24.3, and presenilin 2 (PSEN2) on chromosome lq42.13 (Sisodia and St George-Hyslop, 2002; Tsatsanis et al., 2020). However, it is difficult to establish a relationship between SCD and familial AD genes, which comprise a small portion of early cognitive decline.

The frequency and severity of migraine related to cognitive function remain controversial. Migraine vastly affects cognitive domains, particularly information processing and visuomotor scanning; this (Vuralli et al., 2018) was identified as a significant risk factor for AD and dementia in clinical studies (Gil-Gouveia et al., 2016). Elderly migraineurs tend to report more subjective cognitive symptoms than non-migraineurs; however, the two groups have similar age-related cognitive decline course in 5 years (Martins et al., 2020). No genetic variant of migraine has been associated with Alzheimer’s disease, intelligence, and brain volume by genome-wide association studies (Daghlas et al., 2020). However, the genetic association between SCD and migraine has not been precisely studied. Additionally, whether there are different genetic patterns and symptoms of migraine is unknown. This study investigated additional causative gene loci potentially associated with comorbid SCD and migraine. SNPs, previously identified in a genome-wide association study (GWAS), are potentially associated with SCD and were examined in Taiwanese migraineurs based on subtypes.

## Methods

### Participants

**Figure 1:**
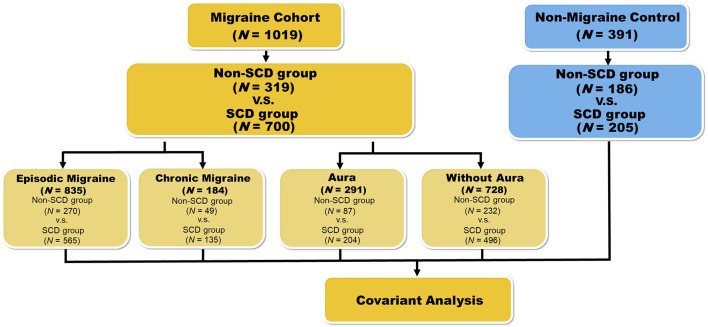
Flowchart representing the workflow for the phenotype association study with variants. For the first phenotype association study, we divided the migraine patients into two groups: non-SCD and SCD. Results are presented in Table 2. In the second round, samples were grouped based on four kinds of conditions: episodic migraine (EM), chronic migraine (CM), aura, and non-aura. The results of these subgroups are represented in Table 3. We also performed a similar analysis on the healthy controls who were also grouped by SCD status. Results are represented in Table 4.

The study enrolled patients with migraine (*n* = 1,019) from the Neurology Outpatient Clinic of Tri-Service General Hospital (TSGH) alongside non-migraine controls (*n* = 392) from the Taipei community through advertisements. Each study participant completed a medical history interview, a neurologic examination, and a battery of neuropsychological tests performed by a board-certified neurologist and headache specialist (FCY). All-migraine and non-migraine groups were subdivided into non-SCD (all-migraine *n* = 319; non-migraine *n* = 186) and SCD (all-migraine *n* = 700; non-migraine *n* = 205) subgroups. Non-SCD/SCD subgroups of the all-migraine group were then stratified into episodic migraine (EM, <15 days per month)/chronic migraine (CM, ≥15 days per month; non-SCD with EM, *n* = 270; non-SCD with CM, *n* = 49; SCD with EM, *n* = 565; SCD with CM, *n* = 135), and migraine with aura (MA)/ migraine without aura (MoA; non-SCD with MA, *n* = 87; non-SCD with MoA, *n* = 232; SCD with MA, *n* = 204; SCD with MoA, *n* = 496; Figure 1).

### Migraine Participants’ Assessment

The diagnosis of migraine was based on the criteria from the third edition of the International Classification of Headache Disorders [ICHD-3; Headache Classification Committee of the International Headache Society (IHS), 2018]. All patients completed a standardized demographic questionnaire, the Migraine Disability Assessment Test (MIDAS; Stewart et al., 2001), and the Hospital Anxiety and Depression Scale (HADS; Zigmond and Snaith, 1983). The clinical features of migraine, such as migraine frequency and duration (years), were also evaluated. The MIDAS consists of a 5-item questionnaire that assesses headache-related disabilities over the previous 3 months and gives a score ranging from 0 to 270 (Stewart et al., 2001). The HADS harbors seven items related to anxiety and depression and has a maximum individual subscale score of 21 (Zigmond and Snaith, 1983).

### SCD Participants’ Assessment

The diagnosis and inclusion criteria for SCD were based on the research criteria for SCD, which include the following: (a) self-reported experience of persistent decline in memory as compared with a previous state (within the past 5 years); (b) performance within the normal range on the Mini-Mental State Examination and the Montreal Cognitive Assessment (adjusted for age, sex, and education); and (c) a score of 0 on the clinical dementia rating (Jessen et al., 2014). All patients completed the SCD questionnaire (SCD-Q; Rami et al., 2014). The SCD-Q includes 24 items assessing three cognitive areas, memory (11 items), language (six items), and executive (seven items) domains; it has a score range of 0–24, with a total SCD-Q score ≥7 reflecting a greater subjective perception of one’s cognitive decline over the past 2 years.

### Evaluation of Non-migraine Controls

The non-migraine control group consisted of volunteers without depression, with or without anxiety disorder, or any psychiatric disorder based on the criteria of the Diagnostic and Statistical Manual of Mental Disorders, Fifth Edition (DSM-V), and with neuropsychological test scores in the normal range. Additional clinical data, such as sex, age, body mass index (BMI), and education level, were also assessed.

### Genotyping and Quality Control

Genotyping was carried out using the Affymetrix Axiom Genome-Wide TWB 2.0 array, which contains 446,000 SNPs representing the Taiwanese genotypic background, with about 105,000 clinically significant SNPs and additional disease-related SNPs added by Thermo Fisher Scientific over the years. Approximately 710,525 SNPs related to drug response, metabolism, and detection of gene copy number variations were evaluated. In August 2019, Academia Sinica’s Taiwan Precision Medicine Initiative reached the milestone of 100,000 samples genotyped using this chip. We applied SNP quality control by excluding SNPs with a call rate of <97% and confirmed the Hardy-Weinberg equilibrium (*P* < 0.00001). Peripheral blood samples from our patients with migraine were collected, and genomic DNA was extracted. Patients’ blood samples were collected in 5-ml EDTA vacutainers (BD, Plymouth, UK). Genomic DNA was extracted using the QIAamp DSP DNA Mini Kit in the QIAsymphony platform (Qiagen, Hilden, Germany). Then, DNA quality was measured with a NanoDrop One spectrophotometer (Thermo Fisher Scientific, Waltham, MA, USA). The signal CEL files generated from Axiom TWB 2.0 SNP array were transformed to genotyping data (tped and tfam) using Genotyping Console made by Affymetrix.

### Statistical Analysis

To evaluate the association between migraine and SCD, phenotype association studies were done using PLINK based = group stratification with and without SCD. The P-value and odds ratio (OR) in the phenotype association study were calculated to study the variant relationship by the 1df chi-square allelic test. For the second stratification, all original groups were divided based on four conditions: EM, CM, MA, and MoA, and were tested by phenotype association study during the first stratification. Conversely, to functionally validate candidate genetic variants involved in SCD, we selected one variant, rs7412, based on similar previous studies (Hostage et al., 2013; Chen, 2016) that investigated the APOE gene in the same SNP microarray. Meanwhile, we compared the migraine SCD group with SCD with the non-SCD groups. Sex effect on SCD analysis was further investigated by evaluating the SCD effect on the subset samples by sex in the migraine cohort and non-migraine controls. Finally, the significant variants were retrieved with a P-value lower than 1E-06 and were annotated based on the RefSeq database (O’Leary et al., 2016) using ANNOVAR (Wang et al., 2010). The correlation between SNPs and the other indices, including migraine features (age, sex, frequency, and severity) and neuropsychiatric comorbidities (insomnia, cognition, depression, and anxiety), was evaluated in migraineurs and non-migraineurs with and without SCD by multivariate regression.

## Results

### Patient Demographic Characteristics

Table 1 represents the comparison of the demographic data of the migraine patients relative to the association between migraine and SCD. There were no significant differences in factors, such as aura/without aura, EM/CM, migraine frequency, sex, and MIDAS score between the groups. However, the other indices in Table 1 yielded significant differences for the all-migraine patients (*P* < 0.05). For the non-migraine group, a similar analysis of the demographic data revealed no significant differences in sex, BMI, or education level (Table 1).

**Table 1:** Patients’ clinical demographic characteristics^a^.

**Section**	**All-migraine**	**All-migraine**		**P-value (All-migraine)**	**Non-migraine**	**Non-migraine**		**P-value (Non-migraine)**
		**Non-SCD**	**SCD**			**Non-SCD**	**SCD**	
Cohort	1,019	319	700	-	391	186	205	-
MA/MoA	291/728	87/232	204/496	0.55	-	-	-	-
EM/CM	835/184	270/49	565/135	0.14	-	-	-	-
Migraine frequency	7.11 ± 7.17	6.57 ± 6.94	7.36 ± 7.27	1.00E-01	-	-	-	-
Migraine duration (years)	26.63 ± 17.84	24.35 ± 17.08	27.68 ± 18.1	4.75E-03	-		-	-
Sex (male/female)	231/773*	66/248	165/525	0.33	200/185*	101/81*	99/104*	0.19
Age (years)	46.45 ± 14.12	43.25 ± 14.80	48.23 ± 13.52	**5.47E-07**	55.14 ± 10.70	53.58 ± 9.38	56.56 ± 9.31	7.64E-03
Body mass index	23.62 ± 4.19	22.97 ± 3.83	23.92 ± 4.31	4.82E-04	25.07 ± 4.76	25.45 ± 38.2	24.72 ± 5.15	1.34E-01
Education (years)	13.85 ± 3.12	14.37 ± 2.88	13.62 ± 3.19	2.18E-04	13.01 ± 3.07	13.24 ± 2.82	12.80 ± 3.22	1.61E-01
MIDAS score	19.16 ± 16.81	18.44 ± 16.58	19.49 ± 16.92	3.65E-01	-	-	-	-
SCD duration (years)	-	-	3.26 ± 3.58	-	-	-	4.10 ± 4.06	-
SCD questionnaire (SCD-Q)	9.84 ± 2.42	7.07 ± 1.08	11.10 ± 1.70	**6.46E-238**	8.97 ± 2.26	7.01 ± 2.27	10.75 ± 1.43	**8.22E-99**
HADS anxiety score	7.64 ± 4.16	6.61 ± 4.06	8.12 ± 4.12	**6.34E-08**	4.91 ± 3.56	4.17 ± 3.43	5.59 ± 3.45	9.38E-05
HADS depression score	6.25 ± 4.11	4.51 ± 3.54	7.05 ± 4.11	**3.05E-22**	4.99 ± 3.63	4.10 ± 3.47	5.80 ± 3.54	3.67E-06

Abbreviations: EM, episodic migraine; CM, chronic migraine; MIDAS, Migraine Disability Assessment Scale; HADS, Hospital Anxiety and Depression Scale. *The sum of the value does not match the total number because of missing data; ^a^P-value was determined by Fisher’s exact test and the t-test grouped by SCD (Subjective cognitive decline) and Non-SCD. Bold values means significant findings due to its *P*-value below 1E-07.

### Association With SCD in the All-Migraine Cohort

**Figure 2:**
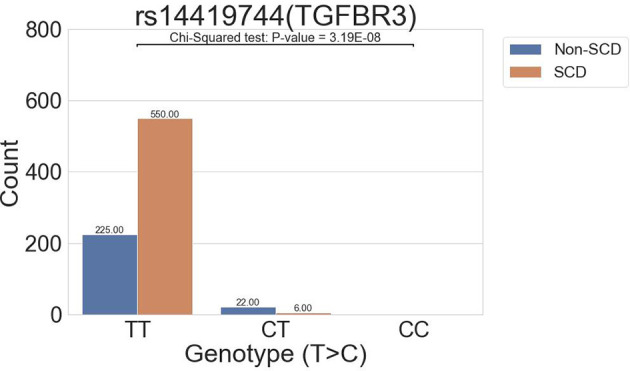
The distribution of the rs14419744 (TGFBR3) variant allele frequency.

The all-migraine cohort was stratified according to SCD status. In the association analysis, one intronic variant was identified in the SCD grouping of the all-migraine patients as having a P-value of <1E-06 (Table 2; Figure 2). The allele frequency of the intronic variant rs144191744 (*P* = 3.19E-08) was 4.45% in the non-SCD group and 0.54% in the SCD group (odds ratio = 0.12). Non-SCD patients had a higher risk allele frequency than SCD patients.

**Table 2:** Association between all-migraine patients, EM, CM, aura, and without aura by SCD grouping^a^.

**Groups**	**SNP**	**Position (GRCh38.p12)**	**MAF**	**TWB**	**Gene**	**Type**	**Variant change**	**Variant allele frequency**		**OR**	**P-value**
								**SCD**	**Non-SCD**		
All-migraine	rs144191744	chr1:91730849	0.02	0.02	TGFBR3	intronic	T>C	0.54%	4.45%	0.12 [0.05, 0.29]	**3.19E-08**
EM	rs144191744	chr1: 91730849	0.02	0.02	TGFBR3	intronic	T>C	0.55%	4.79%	0.11 [0.04, 0.30]	**1.34E-07**
CM	rs4488224	chr11: 87510788	0.43	0.43	LOC107984361	intronic	G>A	30.69%	65.79%	0.23 [0.13, 0.40]	**1.05E-07**
	rs17111203	chr1:94180440	0.066	0.10	ARHGAP29	intronic	A>G	5.94%	27.63%	0.17 [0.08, 0.36]	**6.24E-07**
MoA	rs112400385	chr8:52277062	0.12	0.17	ST18	intronic	T>C	11.88%	23.51%	0.44 [0.32, 0.60]	**3.13E-07**
	rs144191744	chr1:91730849	0.02	0.024	TGFBR3	intronic	T>C	0.49%	4.60%	0.10 [0.03, 0.31]	**7.69E-07**

*Abbreviations: EM, Episodic Migraine; CM, Chronic Migraine; MAF, the minor allele frequency of East Asian group in dbSNP; TWB, Allele frequency in Taiwan Biobank. ^a^A phenotype association study was performed on the all-migraine cohort of the SCD group, which was then was grouped based on four conditions: EM, CM, aura, and without aura. Significant variants with P-values <1E-6 are listed with the allele frequency and odds ratio (OR)*. *Bold values means significant findings due to its *P*-value below 1E-07*.

The distribution of sex differences in the migraine cohort is shown. We further analyzed the association between sex and migraine with SCD. Supplementary Table 4 shows that only four variants in the female group of the migraine cohort could be retrieved. The variant rs144191744 (*P* = 9.58E-07) was also reported in the migraine with SCD cohort (Table 2), suggesting its association with sex.

### Associations Among the EM/CM Subgroups

**Figure 3:**
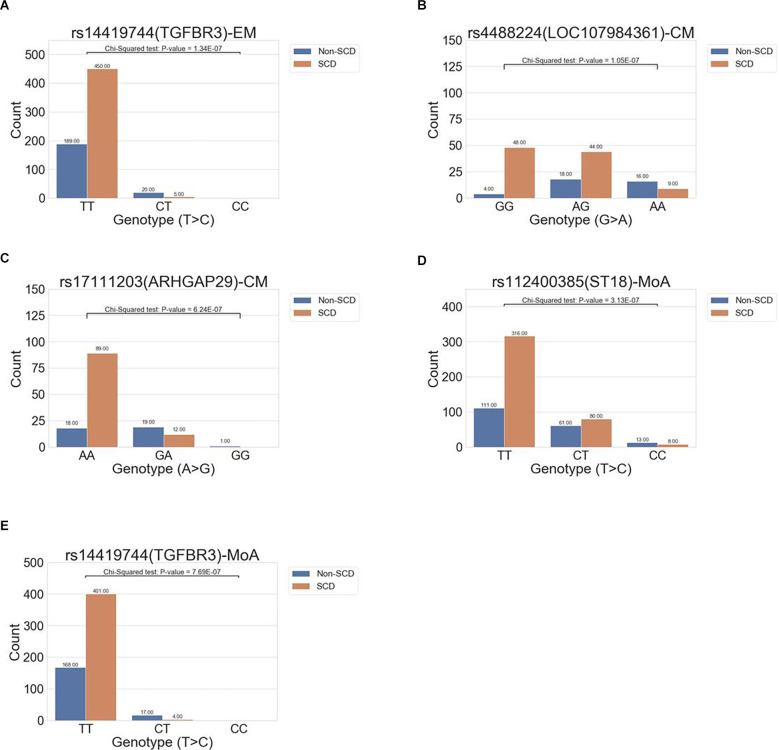
The distribution of allele frequencies of variants across the EM, CM, and MoA subgroups. The study identified genes correlated with SCD that were associated with episodic migraine (rs14419744 in TGFBR3, **A**), chronic migraine (rs17111203 in ARHGAP29, rs112400385 in ST18; **B,C**), and migraine without aura (rs112400385 in ST18 and rs144191744; **D,E**).

In the all-migraine cohort, we identified only one SNP in the EM subgroup and two in the CM subgroup, using the cutoff criteria of *P*-value <1E-06 (Table 2; Figure 3A). In the EM subgroup, the intronic variant was the same as what we found in the all-migraine SCD grouping (Table 2). The risk allele frequency of SNP rs144191744 was 4.79% in the non-SCD group and 0.55% in the SCD group, respectively (*P*-value = 1.34E-07). Conversely, we did not detect the rs144191744 variant in the CM subgroup. We found that, for the rs4488224 and rs17111293 variants in our samples (Table 2; Figures 3B,C), the variant allele frequency in the non-SCD group was higher than in the SCD group.

### With or Without Aura Subgroups Analysis

We further compared migraine groups with aura to those without aura. In the aura group, no variants could be detected using our criteria (i.e., a P < 1E-06). However, we found two intronic variants in the without aura group, and one of the variants, rs144191744, was the same as the variant in both the all-migraine cohort and the EM subgroup [*P* = 7.69E-07 and odds ratio (OR) = 0.10 (0.03, 0.31)] (Figure 3D). According to the variant allele frequency and OR, the pattern was very close to that found in the all-migraine cohort and EM subgroup (Table 2). Another variant, rs112400385, had a variant allele frequency of 23.51% in the non-SCD group and 11.88% in the SCD group (Figure 3E).

### Non-migraine Control Study

In the non-migraine controls, we performed an association analysis to identify the variants associated with SCD with *P* < 1E-06. As shown in Table 3, two variants were significantly associated with SCD, rs2352564, and rs6089473 (*P* = 4.85E-06 and *P* = 8.28E-06, respectively). In the annotation of the two variants, rs2352564 is located in the intergenic region, which has not been fully characterized in the human genome.

**Table 3:** Association between all non-migraine controls grouped by SCD group^a^.

**SNP**	**Position (GRCh38.p12)**	**MAF**	**TWB**	**Gene**	**Type**	**Variant change**	**Variant allele frequency**		**OR**	**P-value**
							**SCD**	**Non-SCD**		
rs2352564	chr7:138232618	0.079	0.13	None	intergenic	A>G	8.20%	20.18%	0.35 [0.22, 0.63]	**4.85E-06**
rs6089473	chr20:61401073	0.44	0.46	CDH4	intronic	T>C	37.98%	54.79%	0.51 [0.37, 0.68]	**8.28E-06**

*Abbreviations: MAF, the minor allele frequency of East Asian group in dbSNP; TWB, Allele frequency in Taiwan Biobank. ^a^All-migraine patients were grouped based on SCD status and compared using PLINK. The significant variants were listed by the empirical *P* < 1E-6 cutoff, with the allele frequency, odds ratio (OR), and 95% confidence interval*. *Bold values means significant findings due to its P-value below 1E-06. There were no SNPs by P-value below 1E-07*.

SNP distribution between the migraine SCD group and non-migraine SCD controls was compared to investigate the effect of SCD on migraine and non-migraine groups (Supplementary Table 1). The results showed only one variant, rs16949672 (*P* = 6.43E-07), with more allele frequency in non-migraine controls than in the migraine SCD group.

Moreover, the association between SCD in the migraine cohort and non-SCD in the non-migraine control (Supplementary Table 2) was also evaluated. One variant, rs79817759, was found to be significantly associated (*P* = 4.44E-08). However, in this case, the trend of the odds ratio was the same as in the previous analysis. In non-migraine controls, the sex effect on non-migraine SCD was further evaluated. However, no significantly associated variants were found because of the sample sizes.

### Replication Study

To assess the variant pattern described in previous SCD studies and to determine whether the variant was associated with other diseases similar to SCD, we chose loci related to SCD (rs7412 and rs429358, the latter one from two SNPs in the TWB2 SNP arrays) to test in our phenotype association studies in the all-migraine patient cohort (Table 4). However, the P-value was not significant. An association study was performed on the non-migraine group, and two variants associated with *P* < 1.0E-06 were identified. Using the two variants to trace the variant profiles in the migraine patient data, variant rs6089473 revealed a trend towards a certain degree of association in the migraine cohort (*P* < 0.05).

**Table 4:** Replication of findings from previous studies^a^.

**SNP**	**Position (GRCh38.p12)**	**MAF**	**TWB**	**Gene**	**Type**	**Variant change**	**Variant allele frequency**		**OR**	**P-value**	**Source**
							**SCD**	**Non-SCD**			
rs7412	chr19:44908822	0.07	0.07	APOE	exonic	T>C	4.17%	9.38%	0.42 [0.089, 1.99]	0.2618	Chen (2016)
rs6089473	chr20:61401073	0.44	0.46	CDH4	intronic	T>C	57.29%	37.5%	2.24 [0.42, 5.09]	0.052	Normal Controls
rs2352564	chr7:138232618	0.079	0.13	None	-	A>G	8.33%	15.62%	0.49 [0.15, 1.63]	0.237	

*Abbreviations: MAF, the minor allele frequency of East Asian group in the dbSNP; TWB, Variant allele frequency in the Taiwan Biobank. ^a^The profiles of variants reported in previous studies were also identified in the all-migraine cohort of the SCD groups*.

We also adjusted the findings from the migraine groups to those from the normal controls. However, the strengths of the association in normal controls were not significant (Supplementary Table 3). The P-values were higher than the threshold (*P* < 1.0E-2). Moreover, some odds ratios were inconsistent with the trend found in the migraine group.

### Multivariate Association Study

We performed multivariate regression analysis using age, sex, migraine frequency, MIDAS, Insomnia Severity Index (ISI), Beck Depression Inventory (BDI), HADS-depression score, HADS-anxiety score, and SCD-Q scores as variables selected by migraine features and neuropsychiatric comorbidities. In the all-migraine cohort, the variant rs144191744 was significantly associated with the SCD group (*p* < 0.001; odds ratio = 5.00; 95% confidence interval = 2.63–9.5; Supplementary Table 5).

## Discussion

In our study, migraineurs harboring SNP rs144191744 (an intronic variant in TGFBR3) were significantly correlated with SCD, especially in the EM and MoA subgroups. Three SNPs, rs112400385 (in ST18), rs4488224 (in LOC107984361), and rs17111203 (in ARHGAP29), were associated with SCD in the migraineurs. In the replication study, non-significant trends with three SNPs: rs7412 (in APOE2), rs6089473 (in CDH4), and rs2352564 (on chromosome 7), were identified. Conversely, two SNPs, rs2352564 and rs6089473, were significantly associated with non-migraineurs with SCD. The findings suggest different pathophysiological and pathogenetic mechanisms of SCD in migraineurs and non-migraineurs.

A GWAS investigating SCD has not been previously performed. Our study attempted to identify marker genes of SCD and their correlation with migraine. To this end, results demonstrated that among the all-migraine cohort, patients with SNP rs144191744 (in TGFBR3) are more likely to be more forgetful. The study identified genes correlated with SCD that were associated with episodic migraine (rs144191744), chronic migraine (rs4488224 in LOC107984361 and rs17111203 in ARHGAP29), and migraine without aura (rs112400385 in ST18 and rs144191744). In addition, two other SNPs were identified in non-migraineurs with SCD (rs2352564 on chromosome 7, rs6089473 in CDH4), and there was a clear trend associated with the underlying pathogenesis of SCD based on migraineur status, as explored below. TGFBR3 is a locus that encodes the transforming growth factor (TGF)-beta type III receptor, which can regulate cell apoptosis (Zheng et al., 2013), and is widely known as a cancer control factor. Additionally, TGF-β participates in the local inflammatory cascade of neurons and glial cells in the brains of patients with AD, which may lead to the overproduction of β-amyloid peptide (Aβ; Bellucci et al., 2007). Recently, several studies have reported that TGFBR3 may be strongly associated with AD. For instance, TGFBR3 gene expression is highly enriched in various key pathways that cause AD, such as amyloid-β formation, regulation of cardiomyocyte differentiation, and actin cytoskeleton reorganization. Furthermore, studies have revealed that certain genes are closely related to AD development, including TGFBR3. The differential expression of TGFBR3 in familial AD and sporadic AD cells was associated with AD severity (Quan et al., 2020)—placing TGFBR3 as a popular candidate gene for AD.

TGF-β is also associated with pain. In inflammation and neuropathic pain models, the TGF-β subfamily protects against the neuroinflammatory response, prevents the destruction of the BBB integrity, facilitates the release of opioid analgesic mediators, and influences the nociception of peripheral sensory neurons in the CNS. Alterations in TGF-β will induce unusual pain in the CNS (Lantero et al., 2012). Thus, TGF-β is highly correlated with inflammation, nerve pain, and cognitive function. The involvement of TGFBR3 in pain mechanisms and cognitive function of migraineurs can be reasonably inferred. We found that one SNP (rs144191744 in TGFBR3) decreased the susceptibility of all migraineurs to SCD. Furthermore, among patients with SCD, EM, or MoA, rs144191744 also showed a high degree of relevance. These findings suggest that rs144191744 is an essential gene to protect migraineurs from SCD, the most representative symptom and transitional stage of AD. To the best of our knowledge, this is the first study to identify potentially related genes. Further research is needed to determine the pathological mechanism of the SCD-related variant in TGFBR3 in individuals with migraine.

In the all-migraine cohort, we identified two SNPs in the CM subgroup. The first variant was rs4488224 (in LOC107984361 on chromosome 11q14.2); however, no genetic study evaluated SNP rs4488224. Another variant was rs17111293 (in ARHGAP29). ARHGAP29 is a gene encoding a specific Rho GTPase protein. Defects in ARHGAP29 may cause non-syndromic cleft lip with or without cleft palate (Leslie et al., 2012). Rho GTPases are known for their roles in cell motility and regulation of cytoskeletal structures and thus play an important role in nerve cells. These are associated with a variety of neurological diseases, such as cognitive defects (ARHGAP15), migraine without aura (ARHGAP28), AD (ARHGAP2), schizophrenia (ARHGAP18), and bipolar disorder (BD; ARHGAP29; Niftullayev and Lamarche-Vane, 2019). In the samples of patients with migraine, 34.3% had a current psychiatric diagnosis, and 73.5% had a lifetime psychiatric diagnosis (Ortiz et al., 2010). Although this study did not screen for other known Rho GTPases genes related to migraine, SCD, or AD, we believe that ARHGAP29 is a potential gene that warrants further in-depth research.

In the migraine without aura group, we identified one SNP associated with the SCD grouping, variant rs112400385 (in ST18). ST18, or the C2H2C-type zinc finger transcription factor, is one of the most abundant DNA binding structures and a member of the neural zinc finger/myelin transcription factor family (Yee and Yu, 1998). ST18 is highly expressed in the brain but is also present in several other tissues, including the heart, kidneys, and liver. The down-regulation of ST18 expression has been shown to lead to changes in pro-inflammatory, pro-, and anti-apoptotic functions in fibroblasts, thereby indicating that ST18 can promote inflammation and apoptosis. Furthermore, ST18 plays a variety of other roles, such as inhibiting breast cancer cells, regulating TNFα (Yang et al., 2008), inducing pancreatic β-cell apoptosis, and impairing insulin secretion (Henry et al., 2014); however, many of its biological functions have not yet been determined.

Moreover, ST18 is highly expressed in oligodendrocytes and plays an important role in myelination. Transcriptional changes in the myelin-forming network are observed in AD, and demyelination is considered a vital AD component. Synaptic cells can directly affect synapse formation and dendritic growth. Additionally, the iron content in oligodendrocytes is associated with the production of the amyloid precursor protein. Iron and other metals make Aβ toxic and are considered the main pathogenic trigger for AD (Kim et al., 2021). Studies have confirmed that ST18 is related to the CDR (Clinical Dementia Rating) score, characterized by a decline in cognitive and functional performance (Humphries et al., 2015). There is limited information regarding ST18, but based on our results and the relationship between ST18 and inflammation and the intensity of ST18 in the brain, we believe that this gene will be a potential marker for migraine and the focus of our future research.

Despite efforts on elucidating the genetic association between migraine and SCD, we identified two SNPs in the non-migraine group (Table 3), which were also replicated in the migraine group (Table 4). The variant rs2352564, an intergenic variant on chromosome 7 without available genetic studies, seems protective of non-migraineurs from SCD [Table 3, OR 0.35 (0.22, 0.63)]. The other variant was rs6089473 in CDH4. CDH4 is a classical cadherin from the cadherin superfamily. Cadherin is expressed in almost all stages of brain development and contributes to CNS regionalization, morphogenesis, and fiber bundle formation in the embryo of spinal animals (Redies, 2000). In the human brain, cadherin is very important for maintaining the structural integrity of the blood-brain barrier (BBB; Li et al., 2011). Aβ enhances the endocytosis of NMDA receptors, resulting in cadherin cleavage of brain vessels, which can exacerbate the development of AD (Daehoon Lee et al., 2018). The variant may lower the risk of SCD in non-migraineurs [Table 3, OR 0.51 (0.37, 0.68)] and increase the risk of SCD in migraineurs [Table 4, OR 2.24 (0.42, 5.09)]. However, all replicating variants, including the rs7412 in the *APOE* gene, did not reach statistical significance in the migraine group (Table 4).

On comparing the migraine SCD group and SCD control group (Supplementary Table 1), one SNP, variant rs16949672 (in MBTD1 on chromosome 17), was identified. No genetic study has evaluated SNP rs16949672. MBTD1, a Putative Polycomb group (PcG) protein-coding gene enriched on embryonic tissues and stem cells in the neural tube and testis as well as on normal tissue in the forebrain, changes the expression of chromatin possibly by methylation of histone H4 (Jacquet et al., 2016). The plasticity of chromatin regulated by the histone code may be related to AD (Bano et al., 2021) and modulate the attack frequency of migraine (Eising et al., 2013). Previously, we found that the *TGFBR3* gene protects patients from SCD under migraine conditions. In contrast, this study showed that the *MBTD1* gene protects patients from migraine under SCD conditions. In addition, when migraine SCD and non-SCD control groups were compared (Supplementary Table 2), one SNP, variant rs79817759 (in LOC283038 on chromosome 10), was identified. However, no genetic study has evaluated SNP rs79817759. The variant rs79817759 seems to protect patients from migraine and SCD. Further studies on the two variants are needed. We also replicated our findings from the migraine SCD group in the normal control group (Supplementary Table 3). The three variants (rs112400385 in ST18, rs1711203 in ARHGAP29, and rs4488224 in LOC107984361) were not associated with SCD in the normal control group. The variant rs144191744 in TGFBR3 was not found in the normal control group. This probably suggests that the *TGFBR3* gene is a risk factor for migraine but protects cognitive function in migraineurs.

We added a sex sub-analysis to compare SCD and non-SCD in the migraine and normal control groups (Supplementary Table 4). Only the female migraine group showed significant findings. The variant rs144191744 in TGFBR3 did not show statistical association in the male migraine and both sexes’ normal control subgroups. It implies that the TGFBR3 gene increases the risk in female but not male migraineurs; however, the result may be influenced by the small sample size of the other three subgroups. Three variants (rs9378123, rs114356017, and rs9404942) located between the *NOTCH4* gene and *TSBP1* and *BTNL2* antisense RNA 1 (TSBP1-AS1) gene on chromosome 6 were identified. The variant rs9378123 was identified as a risk factor for arthropathic psoriasis in the Tree WAS database. There is no genetic study that has evaluated SNP rs114356017. The variant rs9404942 is probably associated with primary biliary cirrhosis (Nakamura et al., 2012). NOTCH4 gene mutation may be associated with migraine (Rubino et al., 2013) and schizophrenia (Takahashi et al., 2003). Notch signaling may be related to AD pathology and neurovascular dysfunction (Kapoor and Nation, 2021). In addition, TSBP1-AS1 gene mutation may be associated with amyotrophic lateral sclerosis (Nakamura et al., 2020), which is related to neurodegeneration and is a spectrum disorder with frontotemporal dementia. Mutation of BTNL2 is a risk factor for sarcoidosis (Valentonyte et al., 2005). Thus, the NOTCH4 gene may increase the risk of migraine and cognitive dysfunction, and the TSBP1-AS1 gene may increase the risk of cognitive dysfunction. These three intergenic variants seem to lower the risk of SCD in female migraineurs by an unknown mechanism. Intergenic suppression, which relieves gene mutation by another compensatory mutation in different loci of the same genome, may explain the fact. Further studies for these three variants are needed.

This study is advantageous because of the well-controlled design of its genetic analysis and its robust statistical analysis. Additionally, qualified doctors were recruited to apply the ICHD-3 [Headache Classification Committee of the International Headache Society (IHS), 2018] and SCD Research Criteria (Jessen et al., 2014) to diagnose the migraine and SCD, following a strictly reviewed protocol. Furthermore, Affymetrix’s Axiom Genome-Wide TWB 2.0 array covers a highly representative sample of the genetic pool in Taiwan.

However, this study has some limitations. First, all study participants were recruited from the neurology clinic of a single medical center, which may limit the generalizability of the findings. Second, the cohort sample is relatively modest. However, these shortcomings can be compensated by using precise diagnostic methods. Further research on larger samples is needed to replicate and expand existing knowledge of genetic risk factors in Asian populations. Further regression analysis of the SCD-Q items score will help realize the influence of SNPs on the clinical characteristics of SCD in migraineurs in future studies.

In conclusion, this study revealed that the SNP rs144191744 (in *TGFBR3*) was significantly correlated with the likelihood of SCD in a cohort of patients with migraine, especially in the EM, MoA, and female subgroups. The variant increases the risk of migraine but protects from SCD. In contrast, the SNP rs16949672 (in *MBTD1*) protects patients from migraine under SCD condition. Furthermore, three SNPs (rs112400385, rs4488224, and rs17111203) are associated with SCD in migraineurs but not with SCD in healthy controls; three (rs9378123, rs114356017, rs9404942) are related to SCD in female migraineurs, two (rs2352564, rs6089473) in healthy controls, and one (rs79817759) non-SCD in healthy controls were identified. Further studies are required to explain the differences in SCD among migraineurs and non-migraineurs in the future.

## Data Availability Statement

The datasets presented in this study can be found in online repositories. The names of the repository/repositories and accession number(s) can be found in the article/Supplementary Material.

## Ethics Statement

The studies involving human participants were reviewed and approved by Tri-Service General Hospital (TSGH) Institutional Review Board. The patients/participants provided their written informed consent to participate in this study.

## Author Contributions

P-KY managed the literature review, conducted the statistical analyses, interpreted the results, and wrote the first draft of the manuscript. C-LT played a major role in data acquisition and revised the manuscript for intellectual content. G-YL, Y-MT, M-CT, and YL interpreted the data and revised the manuscript for intellectual content. C-SL designed the study, provided conceptualization and theory to integrate the findings, and edited the manuscript. Y-KL interpreted the results and provided feedback and comments on the various versions of the manuscript. K-SH provided the conceptualization and theory used to integrate the findings and edited the manuscript. C-KT interpreted the results and provided feedback and comments on the various versions of the manuscript. F-CY designed the study, directed the data collection, provided the overall scientific supervision, interpreted the results, and edited the manuscript. All authors contributed to the article and approved the submitted version.

## Conflict of Interest

The authors declare that the research was conducted in the absence of any commercial or financial relationships that could be construed as a potential conflict of interest.

## Publisher’s Note

All claims expressed in this article are solely those of the authors and do not necessarily represent those of their affiliated organizations, or those of the publisher, the editors and the reviewers. Any product that may be evaluated in this article, or claim that may be made by its manufacturer, is not guaranteed or endorsed by the publisher.
