# Supplementary material for: Genetic Variants Associated With Subjective Cognitive Decline in Patients With Migraine
Source: Front Aging Neurosci. 2022 Jun 15;14:860604. doi: 10.3389/fnagi.2022.860604 (PMC9248861; doi:10.3389/fnagi.2022.860604)
Supplement: Supplementary file 1 [file Data_Sheet_1.PDF]

Supplementary Table 1 Association of the SCD group with the migraine cohort and normal controls

SNP	Position (GRCh38.p12)	MAF	TWB	Gene	Type	Variant Change	Variant allele frequency		OR	P-value
							Migraine SCD	Control SCD		
rs16949672	chr17:51182775	0.01	0.011	MBTD1	intronic	T>C	0.54%	4.08%	0.13 [0.05,0.33]	6.43E-07

Abbreviations: MAF, minor allele frequency of the East Asian group in dbSNP; TWB, allele frequency in Taiwan Biobank.

[a] A phenotype association study was performed on the all-migraine cohort of the SCD group versus SCD in normal controls. Significant variants with P-values <1E-6 are listed with the allele frequency and odds ratio (OR).

Supplementary Table 2 Association of the SCD group in the migraine cohort and non-SCD in normal controls

SNP	Position (GRCh38.p12)	MAF	TWB	Gene	Type	Variant Change	Variant allele frequency		OR	P-value
							Migraine SCD	Control Non-SCD		
rs79817759	chr10:125698189	0.02	0.018	LOC283038	ncRNA_intronic	G>A	1.53%	7.23%	0.20 [0.11,0.38]	4.44E-08

Abbreviations: MAF, minor allele frequency of the East Asian group in dbSNP; TWB, allele frequency in Taiwan Biobank.

[a] A phenotype association study was performed on the all-migraine cohort of the SCD group versus non-SCD in normal controls. Significant variants with P-values <1E-6 are listed with the allele frequency and odds ratio (OR).

Supplementary Table 3 Findings of migraine with SCD in normal controls

SNP	Position (GRCh38.p12)	MAF	TWB	Gene	Type	Variant Change	Variant allele frequency		OR	P-value
							SCD	Non-SCD		
rs112400385	chr8:52277062	0.12	0.17	ST18	intronic	T>C	26.92%	14.29%	2.21 [0.39,12.46	0.3613
rs17111203	chr1:94180440	0.066	0.1	ARHGAP29	intronic	A>G	3.85%	7.14%	0.52 [0.03,9.01	0.6482
rs4488224	chr11:87510788	0.43	0.43	LOC107984361	intronic	G>A	38.46%	35.71%	1.13 [0.29,4.34	0.8641
rs144191744	chr1:91730849	0.02	0.02	TGFBR3	intronic	T>C	0 %	0 %	-	-

Abbreviations: MAF, minor allele frequency of the East Asian group in dbSNP. TWB, Allele frequency in Taiwan Biobank.

[a] All migraine patients were grouped based on the SCD status and compared using PLINK. The significant variants were listed by the empirical P <1E-6 cutoff, with the allele frequency, odds ratio (OR), and 95% confidence interval.

Supplementary Table 4 Sex sub-analysis in the migraine group compared with the SCD and non-SCD groups

Groups	SNP	Position (GRCh38.p12)	MAF	TWB	Gene	Type	Variant Change	Variant allele frequency		OR	P-value
								SCD	Non-SCD		
Female in Migraine	rs9378123	chr6:32229087	0.216	0.21	NOTCH4,TSBP1-AS1	intergenic	A>G	18.69%	31.70%	0.5 [0.38,0.65]	4.37E-07
	rs114356017	chr6:32238145	0.186	0.18	NOTCH4,TSBP1-AS1	intergenic	C>T	16.43%	28.91%	0.48 [0.36,0.64]	5.03E-07
	rs9404942	chr6:32229028	0.19	0.18	NOTCH4,TSBP1-AS1	intergenic	C>T	16.90%	29.12%	0.5 [0.37,0.66]	9.22E-07
	rs144191744	chr1:91730849	0.017	0.024	TGFBR3	intronic	T>C	0.48%	4.38%	0.1 [0.03,0.31]	9.58E-07

Abbreviations: MAF, minor allele frequency of the East Asian group in dbSNP; TWB, allele frequency in Taiwan Biobank.

[a] A phenotype association study was performed on the sex subgroups in the all-migraine cohort and normal controls of the SCD group. Significant variants with P-values <1E-6 are listed with the allele frequency and odds ratio (OR).

Supplementary Table 5 Multivariate regression analysis of variants from the migraine group

SNP	Multivariate	OR	95% confidence level	P-value
	SCD-Q	5.00	2.63-9.5	<0.001
	Age	1.01	1-1.03	0.106
	HADS-Depression	0.91	0.81-1.03	0.146
	Migraine frequency	1.04	0.97-1.13	0.252
**rs144191744**	ISI	1.03	0.97-1.1	0.302
	MIDAS	1.01	0.98-1.04	0.44
	BDI total	0.99	0.94-1.05	0.793
	HADS-Anxiety	1.01	0.91-1.13	0.822
	Sex	0.93	0.49-1.78	0.823

Abbreviations: SCD, subjective cognitive decline; MIDAS, Migraine Disability Assessment Scale; HADS, Hospital Anxiety and Depression Scale; ISI: Insomnia Severity Index; BDI: Beck Depression Inventory.
